# Cellular dissection of the spinal cord motor column by BAC transgenesis and gene trapping in zebrafish

**DOI:** 10.3389/fncir.2013.00100

**Published:** 2013-05-28

**Authors:** Kazuhide Asakawa, Gembu Abe, Koichi Kawakami

**Affiliations:** ^1^Department of Developmental Genetics, Division of Molecular and Developmental Biology, National Institute of GeneticsMishima, Shizuoka, Japan; ^2^Department of Genetics, Graduate University for Advanced Studies (SOKENDAI)Mishima, Shizuoka, Japan

**Keywords:** motor column, zebrafish, Gal4, Mnx, ADAMTS3, gene trapping, BAC

## Abstract

Bacterial artificial chromosome (BAC) transgenesis and gene/enhancer trapping are effective approaches for identification of genetically defined neuronal populations in the central nervous system (CNS). Here, we applied these techniques to zebrafish (*Danio rerio*) in order to obtain insights into the cellular architecture of the axial motor column in vertebrates. First, by using the BAC for the Mnx class homeodomain protein gene *mnr2b*/*mnx2b*, we established the mnGFF7 transgenic line expressing the Gal4FF transcriptional activator in a large part of the motor column. Single cell labeling of Gal4FF-expressing cells in the mnGFF7 line enabled a detailed investigation of the morphological characteristics of individual spinal motoneurons, as well as the overall organization of the motor column in a spinal segment. Secondly, from a large-scale gene trap screen, we identified transgenic lines that marked discrete subpopulations of spinal motoneurons with Gal4FF. Molecular characterization of these lines led to the identification of the ADAMTS3 gene, which encodes an evolutionarily conserved ADAMTS family of peptidases and is dynamically expressed in the ventral spinal cord. The transgenic fish established here, along with the identified gene, should facilitate an understanding of the cellular and molecular architecture of the spinal cord motor column and its connection to muscles in vertebrates.

## Introduction

A comprehensive understanding of how the nervous system generates behavior depends in large part on knowing how the system is organized. With an ever-increasing number of genetic techniques available for visualizing the cellular structures of the nervous system in a wide variety of organisms, our knowledge of the individual neuronal types and their connectivity has been expanding. Among the major challenges which remain is the identification of the functional neural circuits that produce measureable behavioral outputs from the diverse anatomical data and also the genetic programs that assemble these circuits. To achieve this end, it is essential to develop methods for visualizing and manipulating functional neuronal circuits as well as analyzing the gene functions that allow neuronal circuits to operate (Luo et al., 2008; Fenno et al., 2011).

The Gal4-UAS system has been shown to be a useful tool for visualizing and manipulating neural circuits in *Drosophila* (Brand and Perrimon, 1993; Jones, 2009). In this binary gene expression system, the yeast transcriptional activator Gal4 binds to its specific recognition sequence UAS (for upstream activating sequence) and activates the transcription of the target genes downstream of UAS. In zebrafish (*Danio rerio*), targeted expression of Gal4 in specific neuronal populations has been demonstrated using the transgenic technologies based on a meganuclease and transposons (Kawakami et al., 2000; Thermes et al., 2002; Davidson et al., 2003). Of these, the *Tol2* transposon has been extensively applied to gene/enhancer trapping to generate transgenic lines expressing Gal4 in specific neural tissues due to its high transgenesis efficiency (Davison et al., 2007; Scott et al., 2007; Asakawa and Kawakami, 2008; Asakawa et al., 2008). In zebrafish, several different Gal4 derivatives have been tested for stronger and more stable transcriptional activation (Koster and Fraser, 2001; Inbal et al., 2006; Asakawa et al., 2008; Distel et al., 2009; Ogura et al., 2009). In addition, methods of manipulating Gal4 activity using the Gal4 repressor Gal80 (Faucherre and Lopez-Schier, 2011; Fujimoto et al., 2011), morpholino antisense oligonucleotides against Gal4 (Faucherre and Lopez-Schier, 2011; Tallafuss et al., 2012), and the Cre/lox system (Sato et al., 2007) have also been reported. Further, *Tol2*-mediated bacterial artificial chromosome (BAC) transgenesis has been applied to the generation of transgenic fish expressing Gal4 in specific neuronal types (Suster et al., 2009a,b; Bussmann and Schulte-Merker, 2011). With all of these technological advancements and optimizations, the Gal4-UAS system has been applied to a detailed visualization of neuronal morphology with a variety of fluorophores (Sagasti et al., 2005; Aramaki and Hatta, 2006; Hatta et al., 2006; Scott et al., 2007; Arrenberg et al., 2009), monitoring neural activity with fluorescent probes (Dreosti et al., 2009; Del Bene et al., 2010; Muto et al., 2011; Marvin et al., 2013) and manipulating neuronal function with neurotoxin, cytotoxin, and optogenetic probes (Szobota et al., 2007; Asakawa et al., 2008; Douglass et al., 2008; Arrenberg et al., 2009; Del Bene et al., 2010; Janovjak et al., 2010; Yokogawa et al., 2012).

The zebrafish has been a valuable vertebrate model to study neural networks for locomotor behaviors (Saint-Amant and Drapeau, 1998; Budick and O'Malley, 2000; Grillner and Jessell, 2009; McLean and Fetcho, 2011). During locomotion, the motor column of the spinal cord performs the final neural computation prior to the sequential activation of muscles that generates body movement. In zebrafish, the motor column consists of a segmentally iterated set of motoneurons and each segment contains two distinct motoneuron classes: primary motoneurons that are large in size and located dorsally, and smaller secondary motoneurons that are located ventrally (Myers, 1985; Myers et al., 1986; Westerfield et al., 1986). The topographic organization of the motor column has been demonstrated to match the developmental timing of motoneurons and their recruitment pattern during swimming at different speeds (Liu and Westerfield, 1988; McLean et al., 2007, 2008; Gabriel et al., 2011; Menelaou and McLean, 2012).

The development of spinal motoneurons and their connection to the target muscle domains are both under the control of a complex combinatorial network of transcription factors from a variety of different gene families (Jessell, 2000; Shirasaki and Pfaff, 2002; Lewis and Eisen, 2003; Bonanomi and Pfaff, 2010). In zebrafish, the primary motoneurons are produced from a progenitor domain marked by the basic helix-loop-helix (bHLH) olig2 transcription factor in the ventral spinal cord, which is called the pMN domain (Park et al., 2004). Then, the hierarchical interplay between transcription factors from the LIM-homeodomain, as well as the Nkx6 and Mnx homeodomain families, determines the identity of the primary motoneurons (Appel et al., 1995; Tokumoto et al., 1995; Cheesman et al., 2004; Hutchinson and Eisen, 2006; Hutchinson et al., 2007; Seredick et al., 2012). The roles of the transcription factors in the axonal outgrowth of the primary motoneurons have also been reported (Segawa et al., 2001; Liu et al., 2012). On the other hand, the regulatory mechanisms for secondary motoneuron development have largely remained elusive, although several genes, including the LIM-homeodomain protein genes, are known to affect it, when they are mutated or knocked down (Beattie et al., 1997; Cheesman et al., 2004; Pineda et al., 2006; Hutchinson et al., 2007). The availability of transgenic markers for visualizing specific subsets of the secondary motoneurons has also been limited thus far (Meng et al., 1997; Higashijima et al., 2000; Balciunas et al., 2004). Since secondary motoneurons comprise the major cell-type of the motor column, delineating the regulatory mechanisms for secondary motoneuron development, as well as those for primary motoneuron development, is crucial for understanding the motor column organization. Further, since the primary motoneurons have only been described in the anamniote vertebrates to date, cellular and molecular descriptions of the secondary motoneuron should be important to understand the evolution of the motoneurons innervating the axial musculature (Fetcho, 1987, 1992; Tsuchida et al., 1994; Sharma et al., 1998; Agalliu et al., 2009).

Here, we applied BAC transgenesis and gene/enhancer trapping to the dissection of the spinal cord motor column. First, we modified a BAC containing the Mnx-class homeodomain protein gene *mnr2b*/*mnx2b* to establish a transgenic line expressing Gal4FF in a large number of spinal motoneurons. Single cell labeling allowed us to identify diverse types of the spinal motoneurons and to describe both their morphological characteristics and distribution in a spinal segment. Second, we dissected the motor column by means of gene trapping and identified transgenic lines in which specific subpopulations of spinal motoneurons were labeled with Gal4FF. Molecular characterization of one of these lines led to the identification of the ADAMTS3 gene, which encodes an evolutionarily conserved ADAMTS family of peptidases and is dynamically expressed in the ventral spinal cord. The transgenic fish established here, along with the identified gene, provides insights into the cellular and molecular architecture of the motor column and its connection to muscles in vertebrates.

## Results

### Generation of a Gal4FF driver line for the spinal motoneurons using the BAC DNA for *mnr2b/mnx2b*

**Figure 1:**
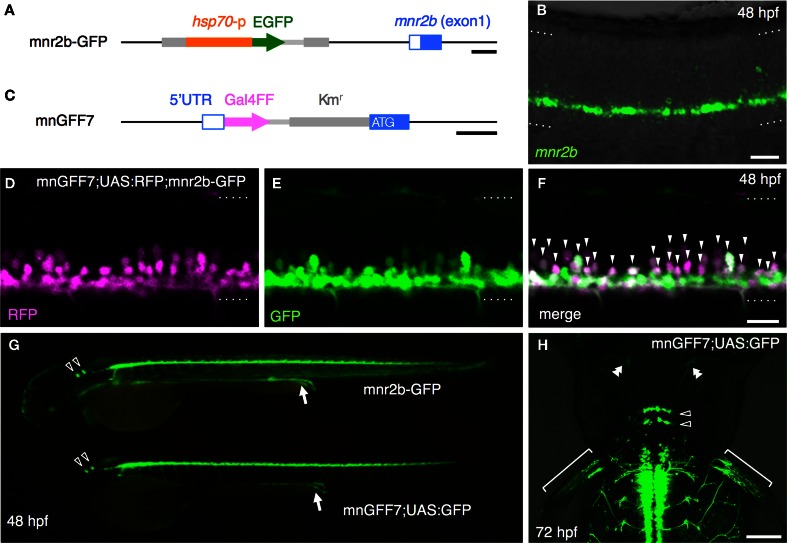
**Construction and characterization of the BAC transgenic line, mnGFF7. (A)** The *mnr2b* genomic locus in the mnr2b-GFP enhancer trap line. The hsp70p-EGFP enhancer trap construct was integrated 1.9 kb upstream of the *mnr2b* gene. **(B)** Expression of *mnr2b* at 48 hpf detected by fluorescent *in situ* hybridization. A confocal section of the spinal cord above the yolk extension in a wild type embryo is shown. The rostral is to the left. **(C)** The *mnr2b* locus on the mnGFF BAC DNA. The PCR-amplified Gal4FF-polyA-Km^r^ cassette is inserted downstream of the 5′UTR of the *mnr2b* (open blue box) by homologous recombination. The coding sequence of exon1 of *mnr2b* (filled blue box) is left intact after the recombination. **(D–F)** The lateral view of the spinal cord of the mnGFF7;UAS:RFP; mnr2b-GFP triple transgenic embryo at 48 hpf. The images shown are the RFP (left), GFP (middle), and merged (right) images of a single confocal slice. The RFP-positive cells whose somata are identifiable are indicated by the arrowheads in **(F)**. The dotted lines demarcate the dorsal and ventral limit of the spinal cord. The rostral is to the left. **(G)** GFP expression in mnr2b-GFP (top) and mnGFF7;UAS:GFP (bottom) embryos at 48 hpf. The arrows indicate the GFP expression in the gut. The open arrowheads indicate the abducens motoneurons. **(H)** The dorsal view of the mnGFF7;UAS:GFP larvae at 72 hpf. GFP is expressed in the abducens and pectoral fin motoneurons. The open and double arrowheads indicate the soma positions and the axon terminals of the abducens motoneurons, respectively. The brackets indicate the axon terminals of the pectoral fin motoneurons. The rostral is to the top. The dotted lines demarcate the dorsal (top) and ventral limit of the spinal cord in **(B,D–F)**. The bars indicate 500 base-pair (bp) in **(A)** and **(C)**, 20 μm in **(B,D,F)**, and 100 μm in **(H)**.

**Figure 2:**
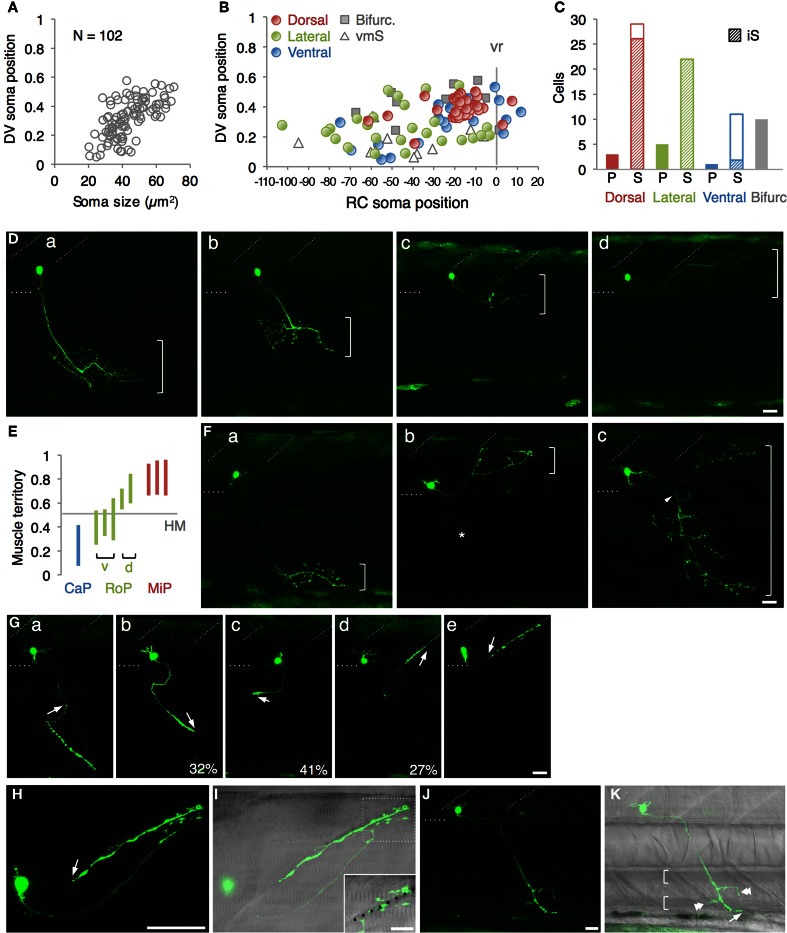
**Single cell labeling of Gal4FF expressing cells in mnGFF7. (A)** The plot of the single GFP-labeled motoneurons (open gray circles) with respect to the soma size on the horizontal axis and to the position of the soma along with the DV axis on the vertical axis. The DV soma position is normalized to the dorsal (*y* = 1) and the ventral (*y* = 0) edge of the spinal cord. **(B)** Distribution of the GFP-labeled cells plotted with respect to the soma position along the RC axis on the horizontal axis and to the DV soma position on the vertical axis. The exit point of the ventral root from the spinal cord is indicated on the RC axis by the gray vertical line marked as vr (*x* = 0). The dorsally, laterally and ventrally projecting cells are shown as red, green, and blue circles, respectively. The cells with bifurcating main axon and the vmS motoneurons are shown as gray squares and open triangles, respectively. **(C)** Cell counts of the neurons innervating the axial muscles based on the axon trajectory and muscle innervation pattern. P and S indicate primary and secondary motoneurons, respectively. Diagonally-striped bars indicate iS-type neurons. “Bifurc” indicates the motoneurons with a bifurcating main axon. **(D)** Primary motoneurons innervating ventrally **(a)**, ventrolaterally **(b)**, dorsolaterally **(c)**, or dorsally **(d)**. **(E)** Muscle innervation territory of the primary motoneurons. The position of the horizontal myoseptum (*y* = 0.5) is indicated as a gray horizontal bar. **(F)** Secondary motoneurons innervating ventrally (**a**, vS), dorsally (**b**, dS), or dorsoventrally (**c**, dvS). The arrowhead in **(c)** indicates the bifurcation in the main axon. The asterisk in **(b)** is the axon of another motoneuron in the opposite hemi-segment. **(G)** The secondary motoneuron innervating the ventral **(a)**, ventrolateral **(b)**, horizontal **(c)**, dorsolateral **(d)**, or dorsal **(e)** myoseptal region. The arrows indicate the axon terminals and roughly indicate their direction of extension. The percentiles show the ratio among the laterally projecting cells. **(H)** A cell innervating the dorsal myotseptum. The arrow indicates the axon terminal. This cell is identical to the one shown in **(Ge)** and the image was taken 6 h after the image **(Ge)** was taken. Note that the morphology of the soma changed dramatically in 6 h. **(I)** The superficial myoseptal region of **(H)** merged with the DIC image. The inset shows a stack of a few confocal sections indicated in the dotted square in **(I)**. The axon collaterals cross the myoseptal boundary marked by the black dotted line. **(J,K)** A vmS-type neuron. The arrow and arrowheads indicate the axon terminal and the collaterals extending along the blood vessels, respectively. All images in **(D–I)** were taken during 72–100 hpf and the rostral is to the left. In **(D,F,G,J)**, the oblique and horizontal dotted lines demarcate the myoseptal boundaries and the ventral edge of the spinal cord, respectively. The brackets roughly indicate the innervation territory along the dorsoventral axis in **(D,F)**, and the axial blood vessels in **(K)**. The bars indicate 20 μm, except in **(I)**, where the bars indicate 10 μm.

We previously established the mnr2b-GFP transgenic line, which labels the spinal motoneurons with GFP by the enhancer trap insertion located upstream of the Mnx-class homeodomain transcription factor gene *mnr2b/mnx2b* (hereafter, *mnr2b*) (Figure 1A) (Asakawa et al., 2012). We found that *mnr2b* was broadly expressed in the ventral spinal cord at 48 hours post-fertilization (hpf) (Figure 1B), consistent with the GFP reporter expression in mnr2b-GFP animals (Figure 2E). Therefore, to establish a Gal4 driver line for the spinal motoneurons, we used a BAC containing the *mnr2b* locus (CH211-172N16), which contains the full coding sequence of *mnr2b* as well as its 55 kb upstream and 78 kb downstream genomic regions. By homologous recombination, we introduced the Gal4FF gene encoding a variant of the yeast Gal4 (Asakawa et al., 2008) immediately downstream of the *mnr2b* 5′UTR (Figure 1C). The resulting BAC construct was named mnGFF. A stable transgenic line carrying mnGFF was established by *Tol2*-mediated BAC transgenesis (Suster et al., 2009b) and designated mnGFF7. We found that the expression pattern of Gal4FF in mnGFF7 line was consistent with that of GFP in the *mnr2b*-GFP line (Figure 1G). Indeed, in the spinal cord of mnGFF7;UAS:RFP;mnr2b-GFP triple transgenic embryos, 97% of the RFP-labeled Gal4FF-expressing cells were also marked by GFP fluorescence at 48 hpf (Figures 1D–F, 116 out of 120 RFP-positive cells from four embryos), suggesting that the mnGFF construct contains most of the *cis*-elements required for *mnr2b* expression in the spinal cord. In mnGFF7;UAS:GFP animals, the GFP signal was also detected in the endodermal and mesodermal tissues, where *mnr2b* is known to be expressed (Wendik et al., 2004), as well as in the abducens and pectoral fin motoneurons (Figure 1H).

### The single cells labelling identified the spinal motoneurons and an interneuron in mnGFF7

In mnGFF7;UAS:GFP larvae at 76–78 hpf, we identified approximately 63 GFP-labeled cells per spinal hemi-segment (62.9 ± 3.2; *n* = 8 hemi-segments at the segment levels 11–13 from three different larvae), which were located within the ventral two-thirds of the spinal cord. The number of the GFP-labeled cells per hemi-segment was significantly higher than the lower limit of the number of axial motoneuron estimated in a previous study (approximately 40 per hemi-segment during day 4–5) (Menelaou and McLean, 2012). These observations prompted us to examine in more detail the GFP-labeled cells in the spinal cord of the mnGFF7;UAS:GFP larvae.

**Figure A1:**
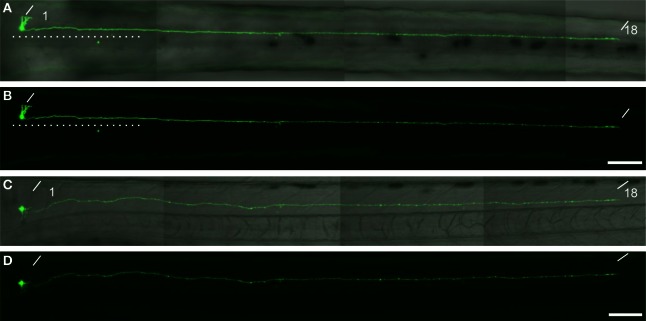
**A descending interneuron identified in the single cell labelling experiment. (A,B)** The dorsal view of a larvae at 76 hpf. The soma of the GFP-labelled cell located at the level of the anterior boundary of somite 1. The axon descended ipsilaterally to the level of somite 18. The dotted lines indicate the position of the midline. (**C** and **D**) The lateral view of the same larva. The oblique lines indicate the anterior boundary of somite 1 and 18. In **(A)** and **(C)**, the DIC image was superimposed onto the GFP image. The scale bars indicate 100 μm in **(B)** and **(D)**.

To investigate the cellular characteristics of individual GFP-labeled cells in the spinal cord of mnGFF7 larvae, we performed a single-cell labeling experiment. The plasmid DNA carrying the UAS:GFP construct was injected into a blastomere of the mnGFF7 embryo at the 4–8 cell stage. To rigorously judge the morphology, individual GFP-positive cells that were uniquely labeled in a hemi-segment were investigated at 72–100 hpf. In total, we observed 103 GFP-labeled single cells (Table 1, *N* = 68 larvae). Of these, 102 cells possessed an axon that exited the spinal cord, indicating that these cells are spinal motoneurons. We found that one out of the 103 cells possessed the soma situated ventrally at segment level 1 and extended its axon ipsilaterally to segment level 18 (Figure A1), suggesting that a small population of descending interneurons is also labeled in mnGFF7 fish.

**Table 1:** **Summary of the cell types identified in the spinal cord of mnGFF7 larvae**.

**Motoneuron**	**Target**	**Type**	**Characteristics**	**Name**	**Cell number (103 cells in total)**
	Axial muscle	Primary	Extensive axonal arborization in the medial muscle. Large, dorsally positioned soma	CaP^*^, MiP^*^, RoP^*^ dRoP^**^, vRoP^**^	9
		Secondary	Extensive axonal arborization in the medial muscle. Specific muscle innervation territory	dS^**^, vS^**^	15
		Secondary	A bifurcating main axon innervating both the dorsal and ventral musculature	dvS^**^	10
		Secondary	Innervating the superficial intermyotomal region	iS^**^	50
		Secondary	No arborization or superficial innervation		5
	Ventromedial area	Secondary	Axon extended toward the ventromedial area close to the axial blood vessels or pronephric duct	vmS^***^	13
**Interneuron**			Ventrally positioned soma with an ipsilateral descending axon		1

* Myers et al., 1986,

** Menelaou and McLean, 2012,

*** *This study*.

### The mnGFF7 line labels the primary and diverse types of secondary motoneurons

The 102 single GFP-labeled motoneurons were found in various positions in a spinal hemi-segment and displayed a typical topographic distribution along the dorso-ventral (DV) axis according to soma size, as determined by their cross-sectional area (Figures 2A,B). Of these, 87% (89 cells) exhibited a main axon innervating axial muscles (Figures 2C–G). The remaining 13% (13 cells) possessed a main axon projecting to the ventral and medial side of the body. The axon terminals and collaterals of this cell type were observed in the vicinity of the axial blood vessels or pronephric ducts (Figures 2J,K). We tentatively named this cell-type “vmS,” for “ventromedial secondary,” because the target tissue(s) of vmS remained to be determined.

**Figure A2:**
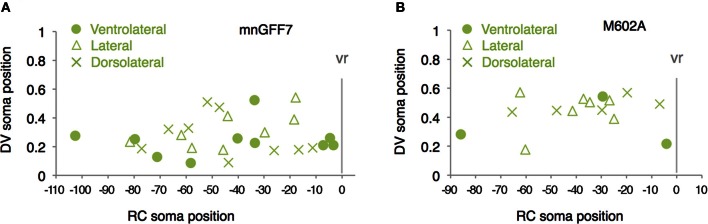
**Distribution of the laterally projecting cells in mnGFF7 (A) and M602A (B) plotted with respect to the soma position along the RC axis on the horizontal axis and to the DV soma position on the vertical axis.** The exit point of the ventral root from the spinal cord is indicated on the RC axis by the gray vertical line marked as vr (*x* = 0). The ventrolaterally, laterally and dorsolaterally projecting cells are shown as circles, triangles, and crosses, respectively.

The 89 cells innervating the axial muscles were categorized into five distinct classes based on their axon trajectories and pattern of muscle innervation. The first class was composed of the cells that displayed a highly extensive axonal arborization that covered a specific territory of the dorsal, lateral, or ventral musculature (10%, 9/89 cells, Figures 2C–E). Based on their larger soma size, dorsal soma positioning and extensive branching of the axon terminal in the medial muscles, these cells likely correspond to the primary motoneurons MiP, RoP, and CaP. The laterally projecting cells were further categorized into the dorsolaterally (dRoP) and ventrolaterally (vRoP) projecting types based on their innervation territory (Figure 2E) (Menelaou and McLean, 2012). The second class was composed of the cells that also innervated the medial muscles, but displayed less branching and had both a smaller soma size and innervation territory, compared to the primary motoneurons (Figure 2F, 17%, 15/89). Some of these correspond to vS (ventrally projecting secondary) and dS (dorsally projecting secondary) (Menelaou and McLean, 2012). The third class was made up of the cells that innervated most of the myotome along the DV axis through a bifurcating main axon (11%, 10/89 cells, Figures 2C,Fc). These cells correspond to the dorsoventrally projecting secondary dvS (Menelaou and McLean, 2012). The fourth class included the secondary motoneurons that possess a main axon extending superficially along the intermyotomal boundary (66%, 50/89 cells, Figures 2C,Ga–e). Of these, the laterally projecting cells could further be classified based on their ventrolateral (32%), horizontal (41%), or dorsolateral (27%) innervation (Figures 2Gb–d). Based on these innervation patterns, these cell types correspond to intermyotomal secondary (iS)-type neurons (Menelaou and McLean, 2012). While the main axons of the class II cells did not cross the myoseptum, their short axon collaterals contacted the myotome structures on both sides (Figures 2H,I), implying that a single iS neuron is able to innervate both of the myotomes that form a myoseptal boundary. The fifth class was composed of the cells that possessed the motor axon but did not exhibit either axonal arborization or superficial innervation, presumably because they were undergoing development (6% 5/89 cells, data not shown). Taken together, these observations demonstrate that the mnGFF7 line is a pan-motoneuronal Gal4 driver that marks the primary motoneurons and diverse types of secondary motoneurons. Interestingly, we noticed that the somas of the dorsally projecting neurons were situated predominantly (84%, 28/33 cells) in the dorsal half of the motor column and clustered within 21 μm of the exit point of the ventral root along the RC axis, mostly on the rostral side, while the ventrally and laterally projecting cells did not display such a bias (Figures 2B, A2A). These observations imply a topographic connection between the GFP-labeled dorsally projecting cells and the dorsal musculature (see Discussion).

### A *Tol2* transposon-mediated gene trap screen for genes expressed in spinal motoneurons

**Figure 3:**
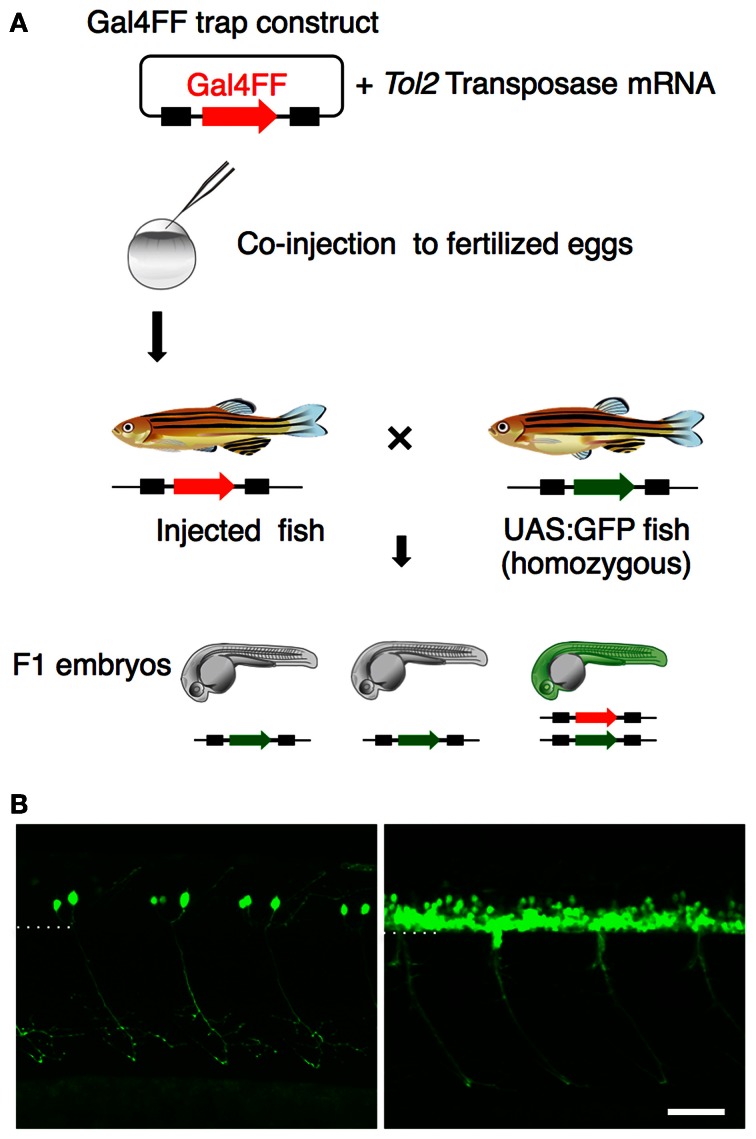
**Gene trapping using the Gal4FF-UAS system. (A)** Scheme for the Gal4FF gene trapping. A plasmid DNA containing a Gal4FF construct and the transposase mRNA were co-injected into fertilized eggs. The injected fish were raised and mated with the reporter fish homozygous for the UAS:GFP insertion. The resulting F1 embryos were analyzed with a fluorescence stereomicroscope. F1 embryos expressing GFP were collected and raised for further studies. **(B)** Examples of GFP expression in the spinal cord and the motor nerve in F1 embryos at 48 hpf. In the left, the primary motoneurons are exclusively labeled. In the right, a large number of GFP labeled cells are observed in the ventral spinal cord. The dotted line demarcates the ventral limit of the spinal cord. The rostral is to the left. The bar indicates 50 μm.

Having identified the various subtypes of spinal motoneurons, we next attempted to identify genetically-defined subpopulations by means of gene trapping. We have been screening transgenic lines expressing Gal4FF in various tissues and cell-types using *Tol2* transposon-based gene/enhancer trap constructs. In these screens (Figure 3A), the wild type fish at the single-cell stage were injected with a plasmid carrying the Gal4FF trap constructs and then raised to adulthood. The injected fish were crossed with homozygous UAS:GFP reporter fish and, for each injected fish, more than 20 F1 embryos were examined for GFP expression at 1 day and 5 days post fertilization. In the ongoing screening with this trap construct, we identified an F1 expressing GFP in the spinal cord approximately every 50 injected fish. These included lines that marked the primary motoneuron selectively (Figure 3B, left), as well as ones that labeled a larger number of spinal motoneurons including the secondary motoneurons (Figure 3B, right).

### The M602A insert predominantly labels the spinal motoneuron with extensive axon arborization

**Figure 4:**
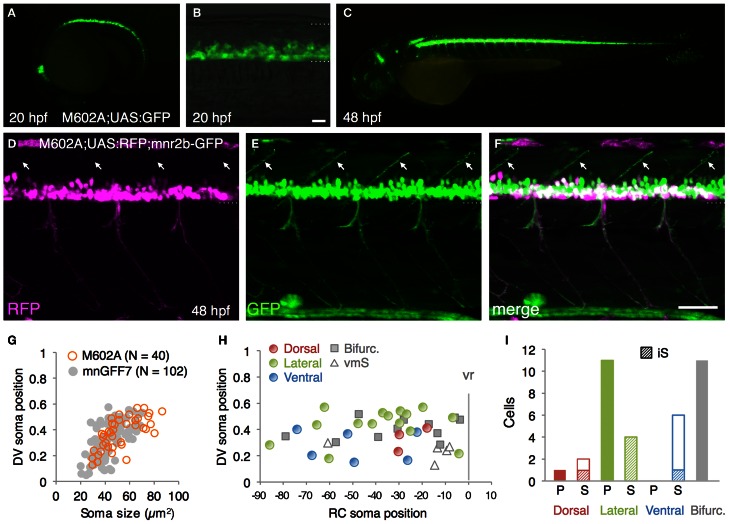
**The spinal motoneurons marked by the M602 insertions. (A–C)** Lateral views of the M602A; UAS:GFP animals. At 20 hpf, GFP expression was detected in the ventral spinal cord and the rostral hindbrain **(A,B)**. The dotted lines demarcate the dorsal and ventral limit of the spinal cord in **(B)**. At 48 hpf, GFP is strongly detected in the ventral spinal cord and the rostral and caudal hindbrain, as well as weakly in other tissues. **(D–F)** Lateral view of the M602A;UAS:RFP; mnr2b-GFP triple transgenic animal at 48 hpf. Arrows indicate the myoseptal region in the dorsal musculature. **(G)** The plot of single GFP-labeled cells (open orange circles, *N* = 40 cells). For reference, the results from the experiment with mnGFF7 in Figure 2A are superimposed as gray circles. **(H)** Distribution of the GFP-labeled cells plotted with respect to the soma position along the RC axis on the horizontal axis and to the DV soma position on the vertical axis. The exit point of the ventral root from the spinal cord is indicated on the RC axis by the gray vertical line marked as vr (*x* = 0). The dorsally, laterally, and ventrally projecting cells are shown as red, green, and blue circles, respectively. The cells with bifurcating main axon and the vmS motoneurons are shown as gray squares and open triangles, respectively. **(I)** Cell counts of the neurons innervating the axial muscles based on the axon trajectory and muscle innervation pattern. P and S indicate primary and secondary motoneurons, respectively. Diagonally-striped bars indicate iS type neurons. “Bifurc” indicates the motoneurons with a bifurcating main axon. The bars indicate 20 μm in **(B)**, and 50 μm in **(F)**.

From the GFP-expressing F1 progeny, we identified gSAIzGFFM602A (hereafter, M602A) fish showing GFP expression in a relatively large number of cells in the ventral spinal cord and motor nerves (Figures 4A–C). In M602A;UAS:GFP embryos, GFP expression was clearly detectable in the ventral spinal cord at 20 hpf (Figures 4A,B), and persisted during the embryonic and larval stages (Figure 4C). GFP expression was also detected in the rostral-most and caudal hindbrain (Figures 4A,C) and very weakly in the dorsal spinal cord. We noticed that the dorsal motor nerve was only weakly stained with GFP in M602A;UAS:GFP animals, while the GFP signal in the lateral and ventral motor nerves were clearly visible (Figure 4C). Consistent with this observation, the dorsally projecting motoneurons innervating the myoseptal region were scarcely labeled with M602A, as judged by *mnr2b*-GFP used as a marker (Figures 4D–F), suggesting that M602A visualizes a subpopulation of the spinal motoneurons.

In order to identify the types of the spinal motoneuron labeled by the M602 insertion, we performed a single cell labeling experiment by injecting the UAS:GFP plasmid and observed individual GFP-labeled cells at 72–96 hpf, as described above. In total, we observed 40 single GFP-labeled cells that possessed an axon that exited the spinal cord (*N* = 31 larvae). The rate of identifying the single GFP-labeled cells was substantially lower in M602A than mnGFF7, most likely due to the early Gal4FF expression in the progenitor cells in M602A. Not surprisingly, we identified other cell types as a single GFP-labeled cell as well, including spinal interneurons and floorplate cells, in M602A larvae.

The 40 single GFP-labeled cells exhibited a topographic distribution pattern along the DV axis according to soma size (Figure 4G). Of these, 35 cells (88%) possessed a main axon innervating axial muscles (Figures 4H,I). The remaining five cells (12%) innervated the ventromedial region of the body, suggesting that they are vmS-type motoneurons (see Figures 2J,K). Interestingly, we found that the neurons innervating axial muscles to be larger in soma size in the M602 larvae (54.5 ± 15 μm^2^, *p* < 0.002) than the mnGFF7 larvae (44.6 ± 12 μm^2^). In addition, the GFP-labeled motoneurons were located in a slightly but still significantly more dorsal side of the spinal cord in the M602A larvae (0.39 ± 0.12, *p* = 0.024) than mnGFF7 larvae (0.34 ± 0.12). Consistent with these observations, the majority of the GFP-labeled cells (83%, 29/35 cells) were primary and secondary motoneurons innervating medial muscles with an extensively arborized main axon in the M602A;UAS:GFP animals (Figure 4I). Among these, cells possessing a laterally projecting axon (*N* = 11/29) and a bifurcating axon (*N* = 10/29) were predominant. The laterally projecting cells innervated a ventral (5/11 cells), lateral (1/11 cells), or dorsal (5/11 cells) subregion of the lateral musculature (Figure 2B). Collectively, these observations suggest that the M602A insertion predominantly labels the motoneurons that are located dorsally in the motor column and have a highly extensive axonal arborization.

### M602A insertion trapped ADAMTS3

**Figure 5:**
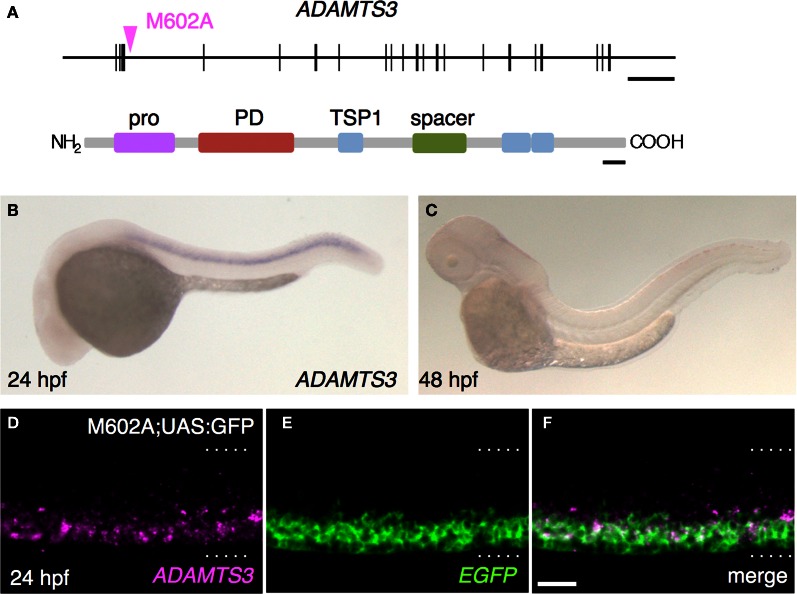
**The M602A insertion traps ADAMTS3. (A)** Structure of the ADAMTS3 locus and the integration site of M602A. M602A is inserted in the 3^rd^ intron of ADAMTS3. The long horizontal bar and vertical bars denote the genomic DNA and the exons of ADAMTS3, respectively. ADAMTS3 consists of multiple functional domains: pro, pro domain; PD, metalloprotease domain; TSP1, thrombospondin type 1 motif; spacer, spacer region [according to Porter et al. (2005)]. **(B,C)** Expression of ADAMTS3 at 24 hpf **(B)** and 48 hpf **(C)**, detected by *in situ* hybridization. **(D–F)** Expression of ADAMTS3 and EGFP detected by double fluorescent *in situ* hybridization. A confocal section of the spinal cord of the M602A;UAS:GFP embryo at 24 hpf was shown. The dotted lines demarcate the dorsal and ventral limit of the spinal cord. The scale bars indicate 20 kbp (top) and 50 amino acids (bottom) in **(A)**, and 20 μm in **(F)**.

In order to identify the gene responsible for the GFP expression in the M602A;UAS:GFP animals, we set out to determine the integration site of the M602A insertion. We cloned the genomic sequences flanking the M602A insertion by adaptor-ligation PCR and found that the insertion was located in a putative gene encoding a protein homologous to the ADAMTS3 protein (a disintegrin and metalloproteinase with thrombospondin motifs3, Figure 5A). The 5′RACE and RT-PCR analysis revealed that the putative gene encoded a 1164 amino-acid protein with multiple functional domains, which was 77% identical to the human ADAMTS3 ortholog (Figure 5A). To analyze the expression pattern of ADAMTS3 in zebrafish, we performed mRNA *in situ* hybridization experiments. The expression of ADAMTS3 was detected in the spinal cord at 24 hpf (Figure 5B). At 48 hpf, the ADAMTS3 expression in the spinal cord was reduced to below a detectable level (Figure 5C), suggesting that ADAMTS3 is dynamically expressed in the ventral spinal cord. The double fluorescent *in situ* hybridization showed that, in M602A;UAS:GFP embryos, the ADAMTS3 and EGFP transcripts were substantially overlapped in the ventral spinal cord (Figures 5D–F). Taken together, these results suggest that the M602A insertion traps the ADAMTS3 gene.

## Discussion

### Cellular organisation of the spinal cord motor column in young zebrafish as viewed through mnGFF7

In the present study, we established the mnGFF7 line, a Gal4FF driver for spinal motoneurons using a BAC DNA containing the *mnr2b* locus sequence. The characterization of the mnGFF7 line disclosed at least three characteristics of the target tissue innervation by the spinal motoneuron that have previously been unreported. First, we found that the dorsally projecting neurons innervating the superficial slow muscle in the myoseptal regions were preferentially identified in the caudal portion of a spinal segment and in the dorsal half of the motor column in mnGFF7 animals. Interestingly, it has been reported that the dorsally projecting neurons with extensive axonal arborization in fast skeletal muscle (dS-type) are typically found in the rostral portion of the spinal segment (Menelaou and McLean, 2012). These two lines of evidence together predict that dorsally projecting secondary motoneurons are topographically positioned along the RC axis and thereby linked to the muscle types they innervate. From the currently available data (Figures 2B, A2A, A2B) (Menelaou and McLean, 2012), such a topographic configuration is not readily apparent for the ventrally or laterally projecting secondary motoneurons. Second, we found that the axon collaterals of single secondary motoneurons innervating the myoseptal regions often crossed the myoseptum, implying that these cell types can make contact with and activate the muscles on both sides of the myoseptum. Similarly, it has been reported that the axons of some secondary motoneurons cross the myoseptum and terminate in the adjacent segment in the adult stage (Westerfield et al., 1986). Such cross-myoseptal innervation would help achieve a near synchronous and unilateral activation of body wall muscles so as to generate coordinated axial movements during the larval, as well as in the adult stage. Finally, in the mnGFF7 larvae, we identified vmS-type motoneurons, which have been only poorly characterized thus far. The observation that axon terminals and collaterals of this cell type were often observable in the vicinity of blood vessels and pronephric ducts raises the possibility that the vmS neurons serve as a part of the autonomic nervous system by directly innervating these tissues or indirectly innervating them via ganglia. These ideas need to be validated in the future by identifying the synaptic targets of vmS neurons.

The establishment of the mnGFF7 line also provided a unique tool to for obtaining insights into the overall organization of the motor column in young zebrafish. In mnGFF7;UAS:GFP larvae, we identified 63 GFP-labeled cells in a spinal hemi-segment on average. Intriguingly, it has been reported that zebrafish on average possesses 65 spinal motoneurons per spinal hemi-segment at the late larval and adult stage (Van Raamsdonk et al., 1983). Therefore, our results raise a possibility that the zebrafish generates spinal motoneurons that are nearly comparable to that in the adult stage, in terms of the total number, over the first 4 days of development. The following two observations, however, suggest that the identity of a fraction of the GFP-labeled cells remains to be determined, and thus precluded an estimation of the number of GFP-labeled spinal motoneuron in mnGFF7;UAS:GFP larvae. First, we identified an interneuron among the 103 GFP-labeled single cells, suggesting that mnGFF7 labels a small population of interneurons. Secondly, the single cell labeling method rarely identified the cells at the two ends of a spinal segment along the RC axis, while we did not observe any such “gap regions” in stable mnGFF7;UAS:GFP or mnGFF7;UAS:RFP double transgenic larvae (see Figure 1D). This is most likely because the transient expression of GFP from the UAS:GFP plasmid tends to label cells with stronger Gal4FF activity, which results in a biased staining compared to the rather uniform labeling achieved with the stable UAS:GFP reporter transgene. In the future, the number of the spinal motoneurons expressing Gal4FF in mnGFF7 fish will be estimated more accurately by retrograde labeling using the photoconversion of the Kaede protein at the ventral root in mnGFF7;UAS:Kaede double transgenic larvae (Kazuhide Asakawa, Koichi Kawakami unpublished observation).

The 102 motoneurons that were analyzed in the single cell labeling experiment displayed a variety of dendritic morphologies: some displayed elaborate branches while others displayed only very tiny ones. The relationship between the dendritic morphologies and the cellular properties we have reported remains a subject for future investigation.

### Genetic dissection of the motor column with BAC transgenesis and gene trapping

Mnx transcription factors are involved in motoneuron differentiation (Tanabe et al., 1998; Seredick et al., 2012). Recently, the zebrafish Mnx genes, *mnx1*, *mnr2a*/*mnx2a*, and *mnr2b*, were shown to be necessary for the specification of the MiP primary motoneuron (Seredick et al., 2012). Our observation that *mnr2b* is expressed rather broadly in the ventral spinal cord at 48 hpf suggests that *mnr2b* is also expressed in the secondary motoneurons and plays a role in their differentiation. An intriguing observation is that *mnr2b* is first expressed transiently in all of the primary motoneurons, and then the expression becomes restricted to the dorsally projecting subtype, MiP (Seredick et al., 2012). Therefore, it would be interesting to determine whether *mnr2b* is expressed in the secondary motoneurons in a similar manner; i.e., *mnr2b* is first expressed in a number of secondary motoneurons and then is restricted to a dorsally projecting subtype. Our observation that the dorsally projecting neurons were the most frequently identified type in the single cell labeling experiment at 72–100 hpf is consistent with this idea. In addition to the motoneuron types labeled in mnGFF7 animals, characterization of those that are labeled with *mnx1* (Wyart et al., 2009; Zelenchuk and Bruses, 2011; Seredick et al., 2012) and *mnr2a cis*-elements will ultimately provide a more comprehensive view of the connections between the axial motoneurons and their target axial muscles established by the Mnx transcriptional program.

We have demonstrated that gene trapping is an effective approach both for identifying genes expressed in a specific population of neurons and analyzing the detailed morphologies of these genetically tagged neurons. We analyzed the gene trap insertion M602A, and identified ADAMTS3 as being expressed in the ventral spinal cord. ADAMTS proteases are known to be evolutionarily conserved non-membrane-bound enzymes that interact with components of the extracellular matrix (ECM) so as to induce degradation (Porter et al., 2005; Salter et al., 2010). It has been demonstrated that ADAMTS3 can process procollagen I and II in cultured cells (Fernandes et al., 2001; Le Goff et al., 2006). Because the M602A insertion primarily labels the motoneurons that have extensive arborization (83%, 29/35 cells), one possible role of ADAMTS3 might be to the promotion the arborization of motor axons through a processing of the ECM. The exact physiological role(s) of ADAMTS3 should be determined by manipulating its function *in vivo*. In the mouse, ADAMTS3 is reported to be expressed in the hindbrain, developing cerebral cortex and dorsal root ganglia, as well as musculoskeletal tissues, but its expression in the spinal cord has not been previously shown (Le Goff et al., 2006). Whether ADAMTS3 function in the spinal cord is evolutionarily conserved also remains elusive at present.

### Toward a functional analysis of the spinal cord motor column with the Gal4-UAS system

Obviously, the Gal4FF driver lines established in the study will be applicable to ectopic expression of various genes and their mutant forms in motoneurons via the Gal4FF-UAS system, and thus should prove to be valuable in the study of motoneuron development, function and disease. Additionally, genetically-encoded functional probes for neurobiology research such as neurotoxins, light-gated ion channels for optogenetics and fluorescent sensors for monitoring neuronal activity can also be delivered to the Gal4FF-expressing motoneurons. Among these approaches, *in vivo* calcium imaging of spinal motoneurons appears promising for motoneuron functional analysis, allowing for the simultaneous visualization of the activity of groups of neurons distributed in the motor column (McLean et al., 2007; Muto et al., 2011, 2013; Warp et al., 2012). We found that when the UAS-regulated calcium indicator GCaMP7a (Muto et al., 2013) was expressed in the spinal motoneurons in mnGFF7 and M602A, the change in fluorescent intensity of the GCaMP associated with body movements was detectable in the cell bodies as well as the axons of spinal motoneurons in larvae. Moreover, the change in fluorescent intensity of the GCaMP was also detectable in pectoral and abducens motoneurons (Kazuhide Asakawa, Koichi Kawakami unpublished observation). Therefore, the mnGFF7 and M602A lines should be applied not only to a functional analysis of axial motoneurons, but also to that of the abducens and limb motoneurons in the near future.

## Materials and methods

### Fish

This study was carried out in accordance with the Guide for the Care and Use of Laboratory Animals of the Institutional Animal Care and Use Committee (IACUC, approval identification number 24-2) of the National Institute of Genetics (NIG, Japan), which has an Animal Welfare Assurance on file (assurance number A5561-01) at the Office of Laboratory Animal Welfare of the National Institutes of Health (NIH, USA).

### BAC engineering and transgenesis

For BAC manipulation, the purified mnr2b-BAC DNA (CH211-172N16, BACPAC Resources Center) was introduced into SW102 *E coli* cells (Warming et al., 2005). Transformation through electroporation was performed as described in (Suster et al., 2009b). For *Tol2* transposon-mediated BAC transgenesis, the iTol2-amp cassette (Suster et al., 2009b) was introduced into the backbone (pBeloBAC11, U51113) of the mnr2b-BAC. The iTol2-amp cassette was amplified by PCR with the primer pair iTol2A-f (5′-gcg cgc caa tag tca tgc ccc gcg ccc acc gga agg agc tga ctg ggt tgC CCT GCT CGA GCC GGG CCC-3′) and iTol2A-r (5′-agc aat ata gtc cta caa tgt caa gct cga ccg atg ccc ttg aga gcc ttA TTA TGA TCC TCT AGA TCA GAT CT-3′), where the lower and upper cases indicate the pBeloBAC11 sequences for homologous recombination and the iTol2-amp annealing sequences, respectively. Two hundred ng of the PCR product (1~2 μl) were used against 25 μl of cell pellet for transformation by electroporation using a Gene Pulser cuvette (0.1 cm, Bio-Rad) and a MicroPulser (Bio-Rad). For introducing the Gal4FF gene into the *mnr2b* locus in the resulting mnr2b-iTol2-amp-BAC, the Gal4FF-polyA-Km^r^ cassette was amplified by PCR using the pKZGFFKm plasmid as the template with the primer pair mnr2b-Gal4FF-f (5′-tat cag cgc aat tac ctg caa ctc taa aca caa caa aag tgt tgc aAT GAA GCT ACT GTC TTC TAT CGA A-3′) and Km-r (5′-ggt tct tca gct aaa agg gcg tcg atc ctg aag ttc ttt gac ttt tcc atC AAT TCA GAA GAA CTC GTC AAG AA-3′), where the lower and upper cases indicate the *mnr2b* sequences for homologous recombination and the pKZGFFKm-annealing sequences, respectively. For BAC transgensis, BAC DNAs were purified with Nucleobond® BAC100 (MACHEREY-NAGEL). A wild-type embryo at the one-cell stage was injected with 45 pg of the purified BAC DNA and 45 pg of *Tol2* transposase mRNA. Germline transmission was confirmed by crossing the injected fish with the homozygous UAS:GFP fish. By crossing sixteen injected fish with the UAS:GFP reporter fish, we identified three founder fish that gave rise to the GFP-positive F1 offspring (germline transmission rate 19%). We chose one of these F1s, named mnGFF7, for further analysis, because it displayed the strongest GFP signal when combined with UAS:GFP at 24 hpf, while the overall GFP reporter expression patterns were indistinguishable among these F1s.

### Single cell labelling

For single cell labeling of Gal4FF-expressing cells in mnGFF7 and M602A larvae, 18pg of pT2KUASGFP plasmid (Asakawa et al., 2008) were injected into a blastomere at the 4- or 8-cell stage. The injected embryos were raised in embryonic buffer containing 0.003% (w/v) *N*-Phenylthiourea (SIGMA, P7629) to inhibit melanogenesis. The cells uniquely labeled with GFP or clearly identifiable as isolated single cells in a hemi-segment were analyzed by confocal microscopy. The GFP-labeled motoneurons at the segment levels 4–20 were analyzed. Soma size, which was defined by measuring the largest cross sectional area of the soma, and location (center of mass) were determined by ImageJ software. Dorsal and ventral limits of the spinal cord were determined based on a DIC image taken with a GFP image. Statistical analyses were performed using Mann–Whitney U tests.

### Gene trapping

Wild type fish were injected with the pT2GgSAIzGFFM plasmid, containing a *Tol2*-based gene trap construct with a splice acceptor from the zebrafish gata6 gene and the codon-optimized Gal4FF gene, and the synthesized mRNA encoding codon-optimized Tol2 transposase (Abe et al., 2011) at one-cell stage, raised and crossed with the UAS:GFP reporter line. Details of pT2GgSAIzGFFM will be described elsewhere (Abe et al. unpublished results).

### Linker-mediated PCR, 5′RACE and RT-PCR

Adaptor ligation-mediated PCR was carried out as described previously (Asakawa and Kawakami, 2009). For RT-PCR and 5′RACE, the total RNA was prepared from 147 wild-type embryos at 48 hpf by homogenizing the embryos in 1 ml of Trizol Reagent (Life Technologies). Five μg of the total RNA were used for cDNA synthesis along with a primer in the ADAMTS3 coding sequence adamts3-ex4r (5′- GAT TGG CTG GTA ATC CAA AGA G-3′) and the 5′RACE System for Rapid Amplification of cDNA Ends version 2.0 (Invitrogen). By using the cDNA as a template, 35 cycles of a first round PCR (94°C for 30 s; 55°C for 30 s; 72°C for 2min) was carried out using the primers adamts3-ex3r1 (5′-TGC AGT CTG TTT TTA GCA ACT CTC-3′) and Abridged Anchor Primer (AAP: 5′-GGC CAC GCG TCG ACT AGT ACG GGI IGG GII GGG IIG-3′) followed by a second round of PCR using adamts3-ex3r2 (5′-CTT GTG ATT GGC TGA TAG AAA ATG-3′) and Abridged Universal Amplification Primer (AUAP: 5′-GGC CAC GCG TCG ACT AGT AC-3′). The 5′RACE products were cloned with a TA cloning kit (Invitrogen) and sequenced. For RT-PCR analysis of the ADAMTS3 gene, the primer pair adamts3-f1 (5′-AGG CCT ACC ATG GTT GTC CTG TCA CTT AGG TTA-3′), and adamts3-r1 (5′-CTC GAG TCA TCT CTC CAC CTC AGA AGA TGT-3′) were used based on the nucleotide sequence for the putative ADAMTS3 (XM_692142). The cDNA obtained from wild type embryos at 48 hpf was used as the PCR template. The cDNA of ADAMTS3 were cloned with a TA cloning kit (Invitrogen) and sequenced (KC894955).

### Whole-mount *in situ* hybridisation

*In situ* hybridization experiments for *mnr2b* and ADAMTS3 were performed against wild-type embryos as described in (Nagayoshi et al., 2008). Antisense probes for *mnr2b* and ADAMTS3 were synthesized with a DIG RNA labeling kit (Roche) using the open reading frame (ORF) of *mnr2b* and the ORF of ADAMTS3 cloned from the RT-PCR analysis as templates, respectively. The ORF of *mnr2b* was cloned by PCR with the primers mnr2b-f1 (5′-ATG GAA AAG TCA AAG AAC TTC AGG-3′) and mnr2b-963r (5′-TCA TAA CCC TGC CCG GAT CTT CCT-3′). The DIG-labeled *mnr2b* and ADAMTS3 antisense probes were detected with a TSA Kit#16 AlexaFluor647 tyramide (T20926, Molecular Probes) and BM Purple AP Substrate (Roche), respectively. The double fluorescent *in situ* hybridization for ADAMTS3 and EGFP was performed against M602A;UAS:GFP embryos as described in (Brend and Holley, 2009). Antisense probes for ADAMTS3 and EGFP were synthesized with DIG RNA labeling kit (Roche) and Fluorescenin RNA labeling kit (Roche), respectively. The DIG-labeled ADAMTS3 and Fluorescein-labeled EGFP were detected with a TSA Kit#16 AlexaFluor647 tyramide and TSA Plus Fluorescein system (NEL74100KT, PerkinElmer), respectively.

### Microscopic analysis

A fluorescence stereomicroscope (MZ16FA, Leica) equipped with a CCD camera (DFC300FX, Leica) was used to observe and take images of GFP-expressing embryos. A live embryo or larva was embedded in 1% low-melting agarose (NuSieve® GTG® Agarose, Lonza) on a Glass Base dish (IWAKI, 3010-035) and subjected to confocal microscopy using an Olympus FV-1000D laser confocal microscope. Images of live embryos and larvae were acquired as serial sections along the *z*-axis and processed with an Olympus Fluoview Ver2.1b Viewer and Adobe Photoshop CS6.

### Conflict of interest statement

The authors declare that the research was conducted in the absence of any commercial or financial relationships that could be construed as a potential conflict of interest.
